# Integrative Genomics Refines Tissues, Candidate Genes and Putative Regulatory Links Involved in the Humic Adaptation of Keystone Freshwater Fish

**DOI:** 10.1111/mec.17698

**Published:** 2025-02-18

**Authors:** M. Yu. Ozerov, K. Noreikiene, K. Taube, R. Gross, A. Vasemägi

**Affiliations:** ^1^ Biodiversity Unit University of Turku Turku Finland; ^2^ Chair of Aquaculture, Estonian University of Life Sciences Tartu Estonia; ^3^ Institute of Biosciences, Life Sciences Center Vilnius University Vilnius Lithuania; ^4^ Swedish University of Agricultural Sciences Drottningholm Sweden

**Keywords:** DEGs, eQTLs, fish, genomics, omics, perch, transcriptome

## Abstract

Although population genomics approaches have been successful in identifying regions of the genome shaped by natural selection, progress in dissecting the molecular mechanisms of adaptive variants and traits has been slow. By integrating multi‐tissue (gill, spleen, olfactory rosette, whole eye, and liver) transcriptomes from 16 wild Eurasian perch ( *Perca fluviatilis* ) populations and previously identified footprints of selection, we prioritise tissues, candidate genes, and putative SNP‐gene expression associations potentially involved in the humic adaptation of this keystone freshwater fish. Over 5000 differentially expressed genes (DEGs) were discovered across the five tissues. A significant excess of outlier SNPs among DEGs found in the gill and spleen tissues indicated their potential involvement in humic adaptation. Next, we identified 2640 *cis‐*eQTLs, and observed significant enrichment of outliers among expression‐associated SNPs (eSNPs) in spleen and olfactory rosette tissues, as well as in all tissues combined. Several eQTLs were found in the regions showing the strongest signals of selection, which also harboured DEGs (chr. 5: *PLAGL2*, chr. 7: *PPP1R8, TCHH*). Thus, our integrative analyses enabled us to pinpoint specific organs that potentially play a key role in adaptation, prioritise candidate genes under divergent selection based on their expression patterns, and identify links between SNPs and transcript abundance variation. We expect that by combining evolutionary and functional genomics perspectives this work provides a practical framework for understanding the genetic basis of phenotypic diversification and adaptation across a wide range of species.

## 1 Introduction

An adaptation to natural, human‐driven, or novel environments in metazoans is expected to alter the functioning of multiple organ systems, resulting in complex changes scattered across the whole genome that reflect past selection at the molecular level. By harnessing the power of high‐throughput sequencing, population genomic approaches have been highly successful in identifying such chromosomal regions shaped by divergent selection (e.g., Foote et al. 2016; Hill et al. 2019; Karlsson et al. 2014; Marques et al. 2017; Ozerov et al. 2022; Roberts Kingman et al. 2021; Vasemägi et al. 2023). Nevertheless, further refinement of candidate genes through the characterisation of putative causal variants, encompassing their molecular function and effect, has been challenging (Cano‐Gamez and Trynka 2020; Kitano et al. 2022; Schaid et al. 2018; Watanabe et al. 2017).

Among the common issues faced during selective sweep mapping is the size of the selective footprints, which often harbour tens to hundreds of genes, making the identification of selection targets difficult (Campbell et al. 2019; Munch et al. 2016; Reid et al. 2021; Roberts Kingman et al. 2021; Wilder et al. 2020). Furthermore, most adaptations have a polygenic genetic basis (Barghi et al. 2020) and many studies to date highlight the importance of regulatory rather than coding sequence variation in shaping organismal phenotypes and driving evolutionary processes in adaptation (Fraser 2013; Jones et al. 2012; Mack et al. 2023; Ozerov et al. 2022; Verta and Jones 2019; Price et al. 2022). Regulation of gene expression is facilitated by two distinct mechanisms: *cis*‐regulatory elements, which primarily influence the expression of nearby genes, and *trans*‐regulatory factors, which modulate the expression of distant genes by interacting with their respective target sequences (Cowles et al. 2002; Mattioli et al. 2020; Wittkopp et al. 2004). Complex interactions of both *cis*‐ and *trans*‐regulatory factors are involved in the regulation of gene expression and both modes can contribute to adaptation in natural populations (Mack et al. 2023; Meiklejohn et al. 2014; Verta and Jones 2019). Yet, predicting the genetic effects on phenotypic variation at different levels of biological organisation has been far from trivial (Amariuta et al. 2023; Brown et al. 2023; Fraser 2013; Henriques et al. 2018; Hernandez et al. 2011; Maurano et al. 2012; Ozerov et al. 2022; Wittkopp and Kalay 2012; Yu et al. 2015). Moreover, phenotypic plasticity and evolutionary change are not mutually exclusive processes (Falconer and Mackay 1996). This is because both genotype and environment interact to shape an individual's phenotype (Pfennig 2021), and this interaction is crucial in driving the variation observed in natural populations. Therefore, to understand the molecular mechanisms of gene expression and its regulation, it is important to study organisms in their native environment.

A key step in understanding the functional role of genomic regions shaped by selection and their phenotypic effects is the integration of omics data obtained from multiple levels, such as whole‐genome, transcriptome, proteome, and metabolome. Recent advancements in genomics have allowed the development of several methods and databases to refine causative genetic variants and facilitate their functional characterisation (Cano‐Gamez and Trynka 2020; Schaid et al. 2018; Uffelmann et al. 2021). These include a diverse set of approaches such as SNP enrichment analyses, fine mapping of SNPs associated with specific traits or diseases (e.g., Finucane et al. 2015; Hu et al. 2011; Pickrell 2014), co‐localisation analyses to explore shared genetic signals across related traits (e.g., Hukku et al. 2021; Nica et al. 2010; Wallace et al. 2012), and transcriptome‐wide association analysis to assess the impact of genetic variants on gene expression patterns underlying complex traits and diseases (Gamazon et al. 2015). However, these approaches are currently constrained by the resolution of existing functional databases and their inability to establish causality (Cano‐Gamez and Trynka 2020; Li and Ritchie 2021). Moreover, most functional databases focus on human diseases, thereby reducing the applicability of these methods in an ecological and evolutionary context. As a result, the genetic and functional underpinnings of adaptive traits for the majority of species remain uncharacterised (Quiver and Lachance 2022, but also see Krishnan et al. 2022; Marand et al. 2023).

Recently, we identified hundreds of candidate regions and genes associated with adaptation to humic environments in the common teleost fish, Eurasian perch ( *Perca fluviatilis* ) by performing whole genome analyses of 32 populations (Ozerov et al. 2022). We observed a significant excess of outlier SNPs in putative regulatory regions (5′UTR, 3′UTR and 5K downstream of genes), which indicates that variation in gene expression likely plays an important role in humic adaptation. In order to refine our understanding of key tissues, physiological processes and genes involved in humic water adaptation, we set out to integrate information from multiple genomic datasets. We hypothesised that the excess of outlier SNPs in differentially expressed genes (DEGs) between humic and clear‐water environments in a specific tissue supports the involvement of identified DEGs in humic adaptation. Conversely, significant underrepresentation of outlier SNPs within DEGs in certain tissues most likely indicates that those tissues play a lesser role in adaptation. However, it is essential to keep in mind that the lack of enrichment of outliers among DEGs can be caused by various factors, including predominance of *trans*‐regulatory changes, mismatch between the sampled developmental stage of an organism and the selection episode or simply lack of power of outlier scans or DE analyses. Furthermore, if a single *cis*‐regulatory change controls a large proportion of variation in an adaptive trait (e.g., *EDA* in sticklebacks, O'Brown et al. 2015), genome‐wide outliers may not show enrichment among DEGs for particular tissue.

To examine the hypotheses described above, we combined data consisting of newly generated perch transcriptomes of five separate tissues (gill, spleen, olfactory rosette, liver, and whole eye) from eight humic and eight clear‐water lakes, and previously identified footprints of selection containing ~10K outlier SNPs and > 3000 candidate genes (Ozerov et al. 2022). By screening multiple tissues with diverse functions in metabolism, sensory perception, immunity, respiration and osmoregulation, and by combining the transcriptomic information with genome‐wide signatures of selection (outlier SNPs), we evaluated (i) whether this integrative genomics approach could assist in highlighting physiological processes most relevant for adaptation. Furthermore, we aimed to (ii) prioritise candidate genes under divergent selection based on their expression differences in five tissues; (iii) identify putative causative links between SNPs and transcript abundance variation by investigating the extent of *cis*‐regulation through detection of eQTLs, and (iv) evaluate whether regulatory SNPs show enrichment of signatures of selection suggestive of their adaptive role; (v) refine the most promising candidate regions and genes by evaluating the overlaps among outliers, DEGs and eQTLs.

## 2 Material and Methods

### 2.1 Target Species

The Eurasian perch ( *P. fluviatilis* ), is a common fish species in the northern latitudes that is able to live in diverse range of environments, such as humic and clear‐water lakes (van Dorst et al. 2019). Humic lakes exhibit higher concentrations of dissolved organic carbon (DOC; Wood et al. 2011), which, in turn, contribute to acidification, affecting the ion composition of the water (Arvola et al. 2010; Erlandsson et al. 2010). The visual environment in humic lakes is essentially “nocturnal” due to the absorption of both down‐welling short‐wavelength light and almost all up‐ or side‐welling light (Eloranta 1978; Jones 1992). Moreover, the oxygen content in humic lakes sharply decreases with depth, leading to hypoxia in deeper areas (Bastviken et al. 2004; Kankaala et al. 2006). In addition to abiotic factors, the high concentration of DOC has a drastic effect on the whole aquatic community, including bacterial, phyto‐ and zooplankton, underwater vegetation and fish species (Blanchet et al. 2022).

### 2.2 Biological Samples

**FIGURE 1:**
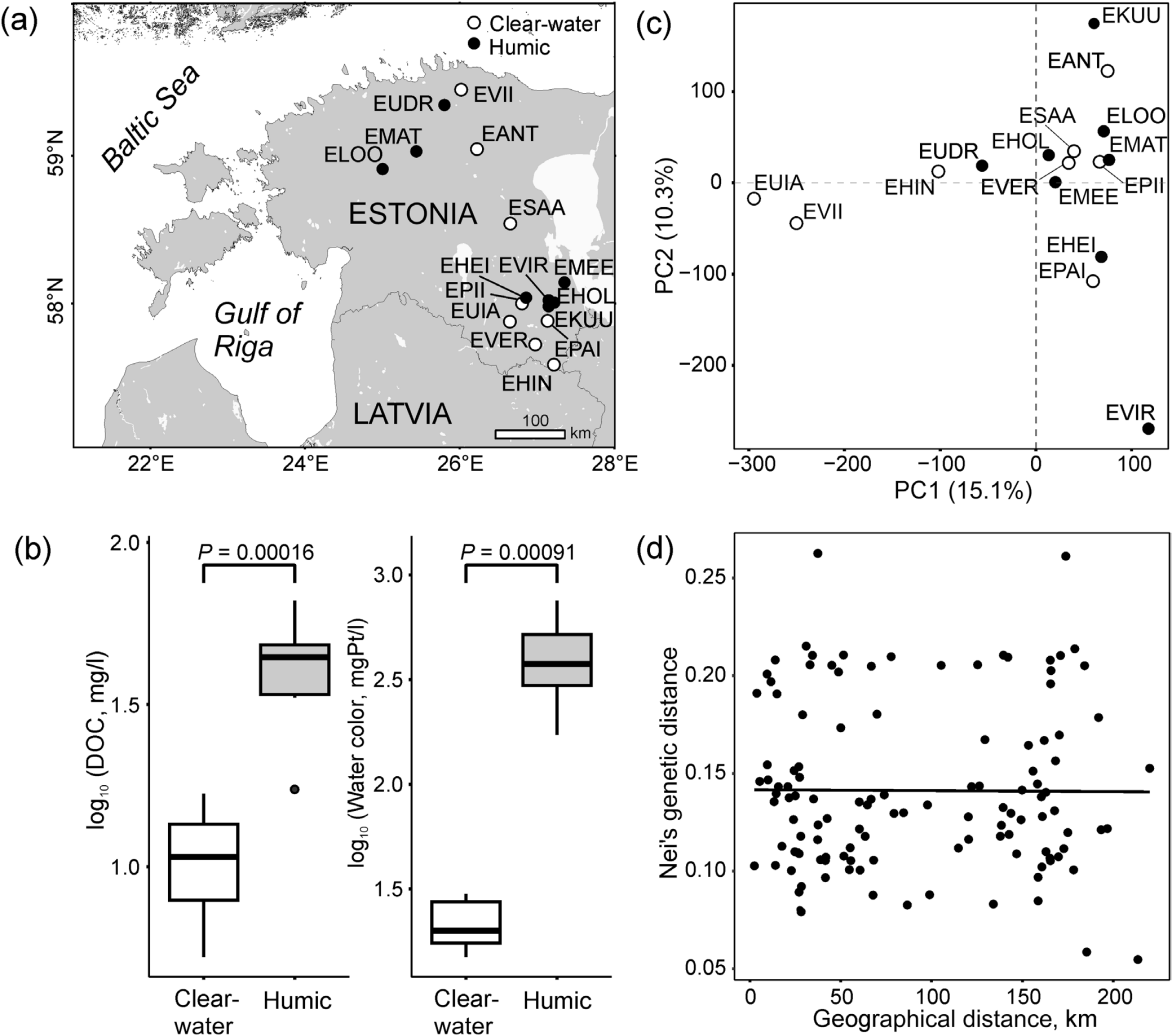
(a) Map indicating sampling locations; perch populations from clear‐water and humic lakes are presented as cricles filled white and black, respectively. (b) Box‐plots showing the level of dissolved organic matter content (DOC) and water colour (log10‐transformed) between 8 humic and 8 clear‐water lakes (*p*‐values of non‐parametric Mann–Whitney *U*‐test are presented). Horizontal line, rectangle, and whiskers indicate the median, 25th and 75th quartiles, and the non‐outlier range, respectively. (c) PCA plot showing relationships among perch populations; populations form clear‐water and humic lakes are presented as circles filled white and black, respectively. (d) Dot plot showing relationships between genetic (Nei 1978) and geographic distances.

In total, 29 and 40 perch were sampled in 2018 and 2021, respectively, from 8 humic and 8 clear‐water lakes in Estonia (Figure 1a, Tables 1, and S1). The selection of lakes for the analysis was based on the drastic differences in water colour (median_HUMIC_ = 381.3 mgPt/l; range = 172.5–752.5 mgPt/l vs. median_CLEAR_ = 20.0 mgPt/l; range = 15.0–30.0 mgPt/l, Mann–Whitney test *p* < 0.001) and DOC (median_HUMIC_ = 44.40 mg/L; range = 17.41–66.10 mg/L vs. median_CLEAR_ = 10.7 mg/L; range = 5.27–16.78 mg/L, Mann–Whitney test *p* < 0.001, Figure 1b). The indexes of water colour and DOC for the studied lakes were retrieved from Ozerov et al. (2022) and Noreikiene et al. (2020). Fish were caught using a gillnet, beach seine or rod. To account for potential differences in sex, we aimed to sample one male and one female from each studied lake. The fish were euthanised by an overdose of ethyl 3‐aminobenzoate methanesulfonate (MS‐222; in 2018) or benzocaine (in 2021) before sampling. Thereafter, total length (to the nearest mm) was measured, and sex was determined by visual examination of the gonads. In 2018, samples of the olfactory rosette were immediately snap frozen in liquid nitrogen. In 2021, the tissue samples of the whole eye, gill, spleen, and liver were placed on dry ice. By screening multiple tissues with diverse functions (gills, as a major hub for respiration and osmoregulation; spleen, as an important reservoir for red blood cells and an organ playing an essential role in protecting the body from pathogens; olfactory rosette, as a chemoreceptor organ, which detects and responds to chemical cues in the water; eye, as an organ responsible for the sense of vision; and liver, playing a central role in metabolism), we aimed to identify physiological processes most relevant to adaptation. The samples were transferred to a −80°C freezer on the day of sampling and were stored there until RNA extraction.

**TABLE 1:** Overview of studied samples and lakes. Lake ID; number of analysed individuals per tissue; location (country, longitude and latitude); environment (humic vs. clear‐water); lake size: Surface area and shoreline length; values reflecting the amount of dissolved organic matter in the lake water: DOC (dissolved organic carbon) and water colour.

Lake	ID	*n*_gill_	*n*_spleen_	*n*_olfactory rosette_	*n*_whole eye_	*n*_liver_	Longitude	Latitude	Ecotype	Lake surface area, ha	Lake shoreline length, km	DOC, mg/L	Water colour, mgPt/L
Äntu Valgejärv	EANT	2	2	2	2	2	59.060	26.240	Clear‐water	1.6	0.47	13.38	17.5
Hino	EHIN	2	2	2	2	2	57.577	27.230	Clear‐water	206.8	11.86	13.91	15.0
Kasaritsa Verijärv	EVER	1	2	1	2	2	57.811	27.047	Clear‐water	25.2	3.71	16.78	27.5
Paidra	EPAI	2	2	2	2	2	57.911	27.191	Clear‐water	11.3	1.76	10.23	30.0
Piigandi	EPII	2	2	1	2	2	58.018	26.791	Clear‐water	44.2	4.39	8.34	20.0
Saadjärv	ESAA	1	2	1	2	2	58.554	26.606	Clear‐water	723.5	19.00	11.24	27.5
Uiakatsi	EUIA	2	2	2	2	1	57.953	26.636	Clear‐water	19.3	2.21	6.68	20.0
Viitna Pikkjärv	EVII	2	2	2	2	2	59.448	26.010	Clear‐water	16.1	2.70	5.27	17.5
Heisri Mustjärv	EHEI	2	1	2	2	1	58.025	26.831	Humic	15.5	2.72	33.28	315.0
Holvandi Kivijärv	EHOL	2	2	2	2	2	58.041	27.198	Humic	6.1	1.52	50.04	520.0
Kuulma	EKUU	2	2	2	2	2	57.957	27.161	Humic	6.4	1.03	47.10	447.5
Loosalu	ELOO	2	2	2	2	2	58.936	25.082	Humic	35.5	2.48	17.41	172.5
Matsimäe Pühajärv	EMAT	2	2	2	2	2	59.061	25.514	Humic	5.5	0.88	41.63	307.5
Meelva	EMEE	2	2	2	2	1	58.141	27.385	Humic	76.0	6.64	47.77	517.5
Udriku Suurjärv	EUDR	2	2	2	2	2	59.370	25.924	Humic	23.0	2.23	34.26	267.5
Virosi	EVIR	2	2	2	2	2	58.026	27.255	Humic	11.3	3.02	66.10	752.5

The collection of samples was conducted in accordance with national legislation based on permits issued by the Estonian Ministry of Environment (no. 10‐1/21/18 and 10‐1/18/29). The requirements outlined in the Annex III (Requirements for establishments and for the care and accommodation of animals) and Annex IV (Methods of killing animals) Section B point 11 of the “Directive 2010/63/EU of the European parliament and of the council of 22 September 2010 on the protection of animals used for scientific purposes” were fully met. The authors have also followed the principles of the 3Rs (Replacement, Reduction, and Refinement).

### 2.3 RNA Extraction, Library Preparation and Sequencing

Before RNA isolation, larger tissues (whole eye, liver, and gill) were mechanically crushed in liquid nitrogen using a custom‐made metal crusher to produce a homogenised powder. Small pieces of the spleen and whole olfactory rosette were homogenised using a Retsch MM400 mill (Retsch). Total RNA was extracted from the homogenised frozen tissues (whole eye, gill, spleen, liver and olfactory bulb; ~ 15–20 mg each) using the NucleoSpin RNA extraction kit (MACHEREY‐NAGEL). The quality of the total RNA sample was evaluated using the TapeStation 2200 (Agilent) electrophoresis and the sample concentration was measured with a Nanodrop ND‐2000 (Thermo Scientific).

The preparation of RNA libraries and sequencing was performed at Novogene, located at the Cambridge Science Park (Cambridge, United Kingdom). Libraries were prepared from total RNA by polyA capture with magnetic beads, followed by reverse transcription to produce cDNA and second strand cDNA synthesis according to the Novogene NGS RNA Library Prep Set (PT042) instructions (Novogene). After fragmentation and end repair, poly‐A tail attachment, adapter ligation, size selection, amplification, and purification, the libraries were sequenced (2 × 150 bp design) using the NovaSeq 6000 (Illumina) instrument.

### 2.4 Read Quality Control and Mapping

Sequencing data were sorted by individuals and indexing adapters were removed at the sequencing facility. The quality of the reads was checked using FastQC ver. 0.11.8 (Andrews 2017). Removal of Illumina adapters, short (< 50 bp) and low quality (average quality score < 25) reads was performed with fastp ver. 0.20 (Chen et al. 2018) using the following parameters: ‐g ‐w 12 ‐r ‐W 5 ‐M 25 ‐trim_front1 9 ‐trim_front2 9 ‐trim_tail1 2 ‐trim_tail2 2 ‐l 50. Further, the filtered sequence reads were mapped to the *P. fluviatilis* reference genome [(Roques et al. 2020) GCA_010015445.1] using hisat2 ver. 2.1.0 (Kim et al. 2015, 2019) with default parameters. Only reads with the best match to their mapped location in the reference genome (primary aligned) were extracted to bam‐files using samtools ver. 1.10 (Li 2011; ‐F 260) for subsequent analyses.

### 2.5 Differential Expression (DE) Analysis

The read counting was performed for exonic gene regions in a non‐strand‐specific manner with the GenomicFeatures ver. 1.46.5 and GenomicAlignments ver. 1.30.0 packages (Lawrence et al. 2013) in R 4.2.2 (R Core Team 2021) using a GFF file containing the *P. fluviatilis* reference genome annotation records. To remove rare transcripts, genes with fewer than 10 raw read counts per tissue in more than 15 individuals were excluded from further analyses.

A Poisson distance matrix was estimated using read counts separately for each tissue with the PoissonDistance function implemented in the PoiClaClu package ver. 1.0.2.1 (Witten 2011) and plotted using classical multidimensional scaling (MDS) with the cmdscale function from the stats package ver. 4.1.3 in R. In total, five samples (one from the gill, one from the spleen and three from the olfactory rosette) were visually detected as outliers on the MDS plots and were excluded from further analyses (Table S1 and Figure S1). Fold changes (FC) in transcript abundance between two groups of perch (from humic vs. clear‐water environment), controlled for geographical location (geographical latitude), fish size (total length) and sex (design = ~Lat.log10 + TL.log10 + Sex + Type), were determined for each tissue with the DEseq2 package ver. 1.34.0 (Love et al. 2014) in R. We also tested an alternative model where population structure was taken into account by adding the two first PCA scores (normalised, see below) as covariates to the model. However, irrespective of the model the main findings were similar to one another and therefore, we present here the results from the first model only. The latitude and total length values were log10‐normalised before the analyses. All genes with an adjusted *p*‐value ≤ 0.05 (Benjamini and Hochberg 1995) were considered to be differentially expressed (DEG) between humic and clear‐water perch.

### 2.6 Enrichment of Footprints of Selection Among DEGs

Outlier SNPs potentially shaped by natural selection driven by humic substances were previously identified by Ozerov et al. (2022) using both population divergence and two genotype‐environment association methods (redundancy analysis (RDA) and latent factor mixed model (LFMM)). In short, the genetic divergence of each SNP locus was estimated as the mean absolute allele frequency difference (*δ*) between humic and clear‐water lakes. Candidate SNPs were identified as SNPs with a *δ* value that was higher than 2.5 SD (standard deviations) from the mean *δ*. RDA and LFMM were performed to test the association of dissolved organic carbon (DOC) and water colour with the estimated allele frequencies. The final set of candidate SNPs potentially under natural selection included 10,245 outlier SNPs, which were identified using at least two methods.

To test if DEGs and nearby gene regions were enriched for outlier SNPs (within 5K up‐ and 5K downstream) potentially shaped by natural selection driven by humic substances (Ozerov et al. 2022), permutation tests were performed using a custom R script for each tissue separately. Specifically, permutation tests compared whether the frequency of outlier SNPs in DEGs was significantly different from that of outlier SNPs in non‐DEGs using 1000 randomly generated subsets without replacement. To test the excess or deficiency of outlier SNPs within DEGs, we randomly shuffled the outlier identity among 810,591 SNPs and evaluated the frequency of permuted outliers within DEGs. This type of randomisation incorporates the differences in SNP density among genes, providing robust support for the association between outlier SNPs and differentially expressed genes.

Further, to test for an excess or deficiency of outlier SNPs among DEGs for each annotation category, i.e., intronic, synonymous, non‐synonymous, 3′UTR, 5′UTR, 5K up‐ and 5K downstream (Ozerov et al. 2022), the frequency of candidate SNPs located in DEGs was compared with that of candidate SNPs located in all expressed genes in a tissue for each annotation category using chi‐squared tests with the stats package ver. 4.1.3 in R.

### 2.7 Identification of SNPs From RNA‐Seq Data

Aligned bam files generated from RNA‐seq reads of four tissues (whole eye, gill, spleen, and liver) were first merged by individual. Genomic variants in the olfactory rosette samples were called separately. Two alternative pipelines were used to identify genomic variants following similar procedures as in Ozerov et al. (2022). First, bcftools ver. 1.16 (Li 2011) mpileup was applied to the locally realigned and sorted BAM files to generate genotype likelihoods with the following SNP calling. Second, the GATK ver. 4.3.0.0 (McKenna et al. 2010) pipeline was applied to call variants using the same BAM files with the following import of single‐sample GVCF files into GenomicsDB using GenomicsDBImport, and final calling of consensus genotypes with GenotypeGVCFs using the same parameters as in Ozerov et al. (2022). Further, genomic variants generated by the two pipelines were filtered using vcftools ver. 0.1.17 (Danecek et al. 2011) applying the following parameters: (i) minimum mean sequencing depth (d) was set to 10; (ii) the consensus quality was ≥ 30; (iii) only bi‐allelic sites were included; (iv) a variant had at least two copies of an allele; (v) a variant did not occur in repetitive genomic regions (vi) minor allele frequency (MAF) was 0.05, and (vii) no missing data were allowed. After applying the quality filters, bcftools and gatk pipelines resulted in generation of 55,854 and 118,787 SNPs among the four tissues samples and 67,724 of 62,378 SNPs among olfactory rosette samples, respectively. Finally, to ensure high quality and reliability of the data, only the variants consistently called by both pipelines were retained: 44,259 and 48,982 SNPs among the four tissues and among the olfactory rosette samples, respectively.

To estimate genetic structure of the 16 studied populations principal component analysis was performed using 44,259 SNPs based on the four tissue samples with the *dudi.pca* function of the ade4 ver. 1.7‐22 package (Dray and Dufour 2007) in R. Genetic relationship among populations was estimated as Nei's genetic distances (Nei 1978) using the *nei.dist* function from the poppr ver. 2.9.6. package (Kamvar et al. 2014) in R. Next, the relationships between genetic and geographic distances, estimated as the shorter pairwise distance between lakes using the *distm* function of the geosphere package ver. 1.5‐19 (Hijmans 2023), were evaluated. In addition, pairwise *F*_ST_ was calculated with the *pairwise.fstb* function of the PopGenReport ver. 3.1 package (Adamack and Gruber 2014) in R.

### 2.8 Identification of *cis*‐Variants Associated With Transcript Abundance

The association between each SNP and each gene expression level was tested with the R package MatrixEQTL (Shabalin 2012). The associations were tested separately for each tissue and only the genotypes and expressed genes of the same individuals within each tissue were included. SNPs with MAF < 0.10 and having only heterozygous genotypes were excluded. eQTLs were identified by modelling the effect of genotype as additive linear (least squares model), accounting for sex, environment type, and the first two normalised PCA scores for population structure, which were included as covariates in the model. Given that the *cis*‐eQTLs are mostly centred around the transcribed region, and to reduce the burden of multiple testing, *cis*‐regulatory region was set as the maximum distance for each gene that spanning 50 Kb upstream and downstream from the gene start and end positions, respectively (Peters et al. 2016; Powell et al. 2012; Stranger et al. 2007; Veyrieras et al. 2008). Gene‐SNP associations were considered significant at FDR ≤ 0.05 (Benjamini and Hochberg 1995). To test for significant enrichment of outliers among identified eSNPs (i.e., SNPs that are significantly associated with gene expression), we used a 2 × 2 chi‐square test. The test compares outlier and nonoutlier count from Ozerov et al. (2022) with the outlier and nonoutlier count among eSNPs using the Yates (Yates 1934) correction.

### 2.9 Gene Ontology (GO) Analysis

Human orthologue gene symbols were searched using complete gene names in the NCBI database. GO enrichment analysis of DEGs against all orthologous genes expressed in each tissue as a background was performed using the gprofiler package ver. 0.2.1 (Kolberg et al. 2020; Raudvere et al. 2019) in R. GO terms with a g:SCS threshold ≤ 0.05 were considered significant.

## 3 Results

### 3.1 Relationships Among Tissues and Samples

**FIGURE 2:**
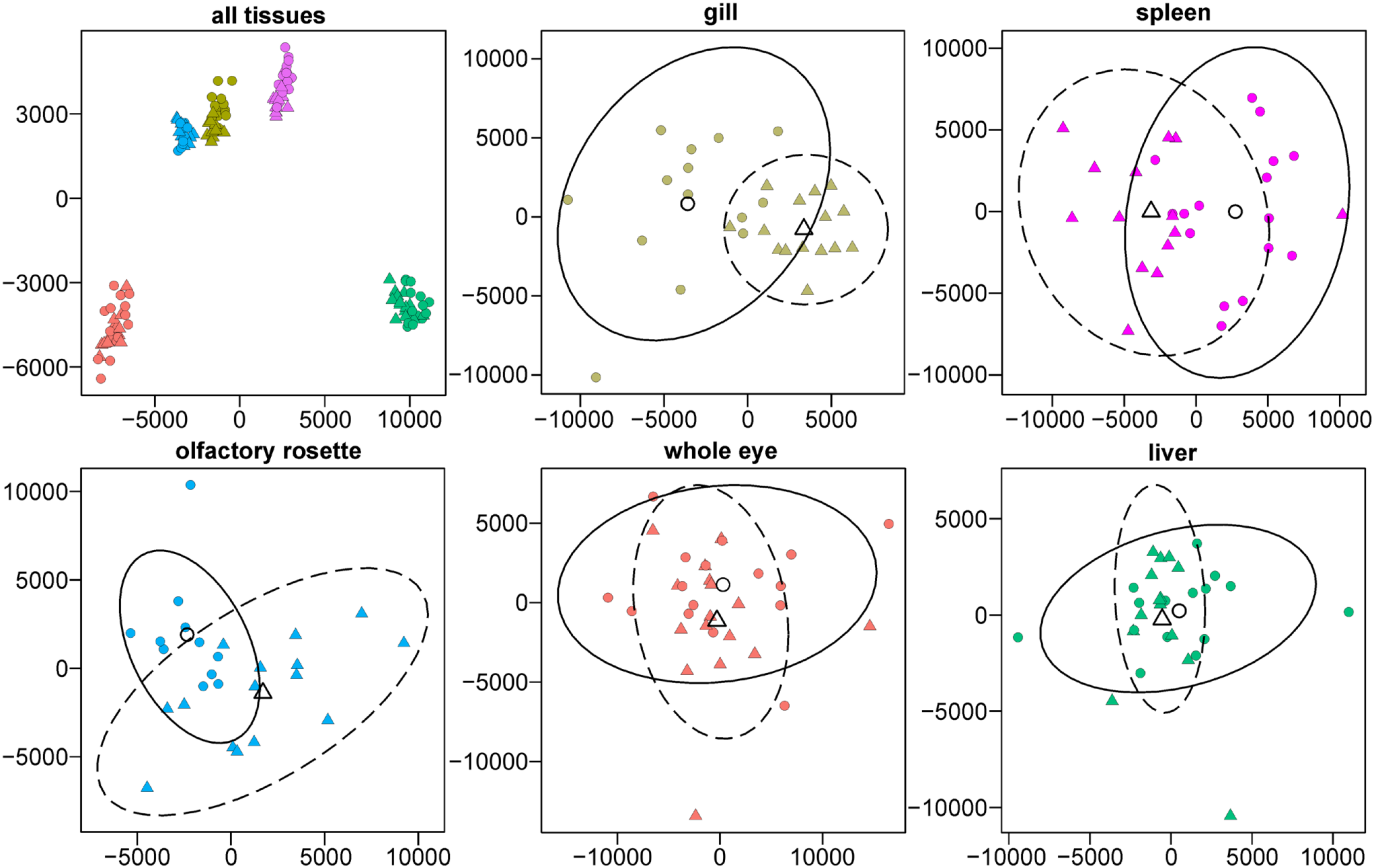
MDS plots showing relationships of all samples analysed, gill (olive green), spleen (magenta), olfactory rosette (deep sky blue), whole eye (light red) and liver (spring green) tissue samples. Clear‐water and humic perch samples are presented as filled circles and filled triangles, respectively. Open circles, open triangles, solid line and dashed line ellipses show the central points and distribution of the clear‐water and humic samples data, respectively.

The number of raw reads ranged from 35.1 to 68.8 M for each tissue sample, totalling 7155.1 M, and an average of 86.4% of the raw reads passed QC and were further mapped to the reference genome. In total, from 15,020 to 20,189 genes per tissue were expressed out of 24,326 annotated protein‐coding genes in perch after applying QC filters (Table 2). A clear separation among tissues was evident on a MDS plot based on 146 samples and all expressed genes (Figure 2), with much larger among‐tissue differences compared to within‐tissue differences between humic and clear‐water perch. The separation between humic and clear‐water perch was more pronounced for the gill, spleen and olfactory rosette samples (Figure 2), whereas the whole eye and liver showed less extensive expression differences between the two habitats (Figure 2). In total, 13,633 genes were commonly expressed in all five tissues, whereas expression of a smaller number of genes (from 143 genes in spleen to 1261 genes in eye) was tissue‐specific (Figure S2).

**TABLE 2:** The number of expressed genes, differentially expressed genes (DEGs) between *P. fluviatilis* from clear‐water and humic environment, and overlap between DEGs and candidate genes (i.e., genes harbouring outlier SNPs within 5K up‐ and 5K downstream area) identified by Ozerov et al. (2022) (candidate DEGs) for five differernt tissues.

Tissue	Expressed genes	DEGs	Upregulated in humic environment	Downregulated in humic environment	Upregulated in humic environment (%)	Downregulated in humic environment (%)	Candidate DEGs	Candidate DEGs (%)
Gill	18,446	4311	1996	2315	46.3	53.7	639	14.8
Spleen	16,685	891	314	577	35.2	64.8	132	14.8
Olfactory rosette	18,980	705	259	446	36.7	63.3	91	12.9
Whole eye	20,189	557	194	363	34.8	65.2	73	13.1
Liver	15,020	241	144	97	59.8	40.2	34	14.1

The studied populations showed strong genetic divergence from each other (pairwise *F*_ST_ = 0.07–0.43; Table S2), reflecting the importance of isolation and genetic drift in shaping population structure. We did not observe pronounced genetic clustering on the PCA plot; the populations did not group according to the environment (Figure 1c). Additionally, there was no evidence for isolation by distance (*r* = −0.007, *p* = 0.939; Figure 1d).

### 3.2 Differential Expression in Five Tissues

**FIGURE 3:**
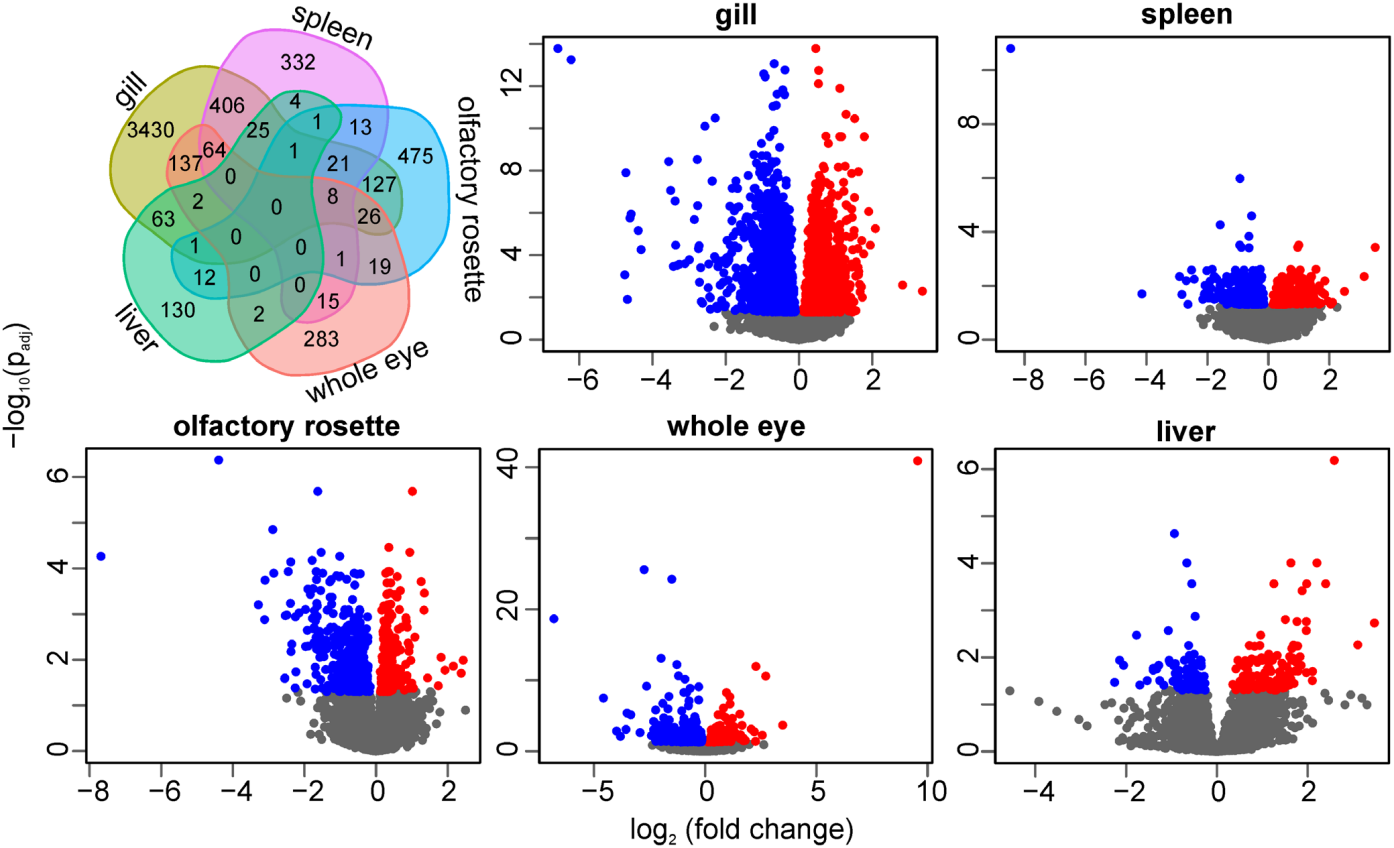
Venn diagram showing the common and unique DEGs among the five tissues (top left corner). Volcano plots showing differentially expressed genes of *P. fluviatilis* between humic and clear‐water environments in the gill, spleen, olfactory rosette, whole eye and liver tissues. Up‐regulated and down‐regulated genes in humic environment are presented as filled red and blue dots, respectively. Non‐significantly differentiated genes are shown as grey dots.

In total, 5598 genes were differentially expressed (*p*_adj_ ≤ 0.05) between perch from humic and clear‐water environment across all tissues (Figure 3, Tables 2 and S3). The largest number of DEGs was observed in gill (*n* = 4311), where 1996 (46.3%) were up‐regulated and 2315 (53.7%) were down‐regulated in humic perch (Table 2), indicating that differences in water chemistry, oxygen content and/or pathogen and parasite community are likely important factors shaping gene expression variation between humic and clear‐water environment. The number of DEGs (*n* = 891) in spleen was nearly five times lower than that in gill, with 314 (35.2%) and 577 (64.8%) genes being up‐ and down‐regulated in humic perch, respectively. In the olfactory rosette 705 DEGs were detected, of which 259 (36.7%) were up‐ and 363 (63.3%) were down‐regulated in humic perch. In the eye 557 DEGs were detected, among them 194 (34.8%) were up‐ and 363 (65.2%) down‐regulated in humic perch. The lowest number of DEGs was observed in liver (*n* = 241), of which 144 (59.8%) and 97 (42.2%) were up‐ and down‐regulated, respectively, in humic environment. In general, the number of down‐regulated DEGs significantly exceeded that of up‐regulated in humic environment (chi‐squared test: *χ* ^2^ = 11.74–39.39, *p* = 1.0 × 10^−5^—6.1 × 10^−4^) in all the tissues, except for the liver, where the number of up‐regulated DEGs was significantly higher (chi‐squared test: *χ* ^2^ = 4.62, *p* = 3.1 × 10^−2^).

In total, 948 (16.9%) DEGs were found in two or more tissues (Figure 3 and Table S3). Eight common DEGs were down‐regulated in four tissues of humic perch (gills, spleen, olfactory rosette and eye: *CRTAC1*, *SOCS1*, *TTN*, *KLHL6*, *LCP1*, *MR1*, *IRF1*, *PLD4*; Figure 3 and Table S3). One common DEG (*IFITM3*) was down‐regulated in the gills, spleen and olfactory rosette in humic environment, whereas it was up‐regulated in the liver. In addition, 1 to 64 DEGs overlapped in three studied tissues (Figure 3 and Table S3). Among the tissue pairs, the largest overlap of DEGs was observed between gill and spleen (*n* = 525), followed by gill and eye (*n* = 237) and gill and olfactory rosette (*n* = 184). None of the DEGs were common for all five tissues.

### 3.3 Functional Enrichment Analysis of DEGs

Gene Ontology (GO) analysis revealed enrichment of multiple GO terms for DEGs in all the studied tissues. The highest number of enriched GO terms was observed in gill: 288 biological process (BP), 42 cellular component (CC) and 17 molecular function (MF) GO terms (Table S4). The most significant terms included signal transduction (BP GO:0007165) and cell adhesion (BP GO:0007155), cell periphery (CC GO:0071944), plasma membrane (CC GO:0005886), signalling receptor binding (MF GO:0005102) and signalling receptor activity (MF GO:0038023). In the olfactory rosette, DEGs were enriched for 76 BP, 17 CC and 5 MF GO terms, of which the most significant included immune response (BP GO:0006955), immune system process (BP GO:0002376), cell periphery (CC GO:0071944), plasma membrane (CC GO:0005886), signalling receptor binding (MF GO:0005102) and immune receptor activity (MF GO:0140375). A similar number of enriched GO terms were observed for DEGs in the liver (75 BP, 10 CC and 1 MF) and spleen (63 BP, 10 CC and 1 MF). Mitotic cell cycle process (BP GO:1903047), mitotic cell cycle (BP GO:0000278), nuclear chromosome (CC GO:0000228), DNA replication preinitiation complex (CC GO:0031261) and single‐stranded DNA helicase activity (MF GO:0017116) were among the most significant GO terms in the liver, while immune system process (BP GO:0002376), immune response (BP GO:0006955), cell periphery (CC GO:0071944), plasma membrane (CC GO:0005886), and enzyme binding (MF GO:0019899) were the most significant in the spleen. The lowest number of enriched GO terms was revealed in the eye (26 BP, 4 CC and 1 MF), with immune response (BP GO:0006955), response to external stimulus (BP GO:0009605), photoreceptor cell cilium (CC GO:0097733), photoreceptor outer segment (CC GO:0001750) and oxidoreductase activity (MF GO:0016491) being the most significant (Table S4).

Enrichment of GO terms among DEGs overlapped in two or more tissues was observed in seven of 26 possible combinations (Table S4). The majority of significant GO terms among overlapped DEGs were linked to immunity, e.g., immune system process (BP GO:0002376) immune response (BP GO:0006955), regulation of immune response (BP GO:0050776), positive regulation of immune system process (BP GO:0002684) and T‐cell activation (BP GO:0042110) were observed (Table S4).

### 3.4 Overlap Between DEGs and Putative Signatures of Selection

The permutation test showed a significant excess of outlier SNPs among DEGs in two tissues, the gill and the spleen (Table 3 and Figure S3). The excess of outlier SNPs among DEGs was also observed when DEGs from all tissues were combined; however, this pattern was likely driven by the excess of outliers in the gill and the spleen. Likewise, the proportion of DEGs harbouring outlier SNPs was higher in the gill and the spleen (14.8% for both tissues), whereas a lower proportion was observed in the olfactory rosette (12.9%) and in the whole eye (13.1%; Table 2).

**TABLE 3:** The number and frequency of outlier SNPs in DEGs and in non‐differentially expressed genes in five tissues.

Tissue	No. of non‐outlier SNPs in DEGs	No. of outlier SNPs in DEGs	Frequency of outlier SNPs in DEGs	No. of non‐outlier SNPs in non‐DEGs	No. of outlier SNPs in non‐DEGs	Frequency of outlier SNPs in non‐DEGs	No. of permuted values larger than observed	*p*
Gill	137,311	2093	0.0150	341,128	4366	0.0126	0	**0.000**
Spleen	27,404	481	0.0172	390,353	5164	0.0131	0	**0.000**
Olfactory rosette	23,712	291	0.0121	449,271	6023	0.0132	916	0.916
Whole eye	14,844	190	0.0126	488,753	6527	0.0132	757	0.757
Liver	6821	66	0.0096	362,489	4962	0.0135	999	0.999

*Note:* The enrichment of outlier SNPs in DEGs was tested using custom permutation test script, significant enrichment marked with bold.

Further comparison of outlier SNPs among DEGs by annotation categories revealed a significant excess of outlier SNPs among 5K upstream gene variants in the spleen, intron variants in the gill and non‐synonymous variants in the eye (Table S5). On the other hand, we also observed a significant depletion of outliers among DEGs for 5′UTR and 5K downstream variants in the gill, and 3′UTR variants in the eye. Similarly, a significant depletion of outlier SNPs among DEGs was observed for 5K downstream variants when the data from all tissues were combined (Table S5).

### 3.5 *cis*‐eQTLs

**FIGURE 4:**
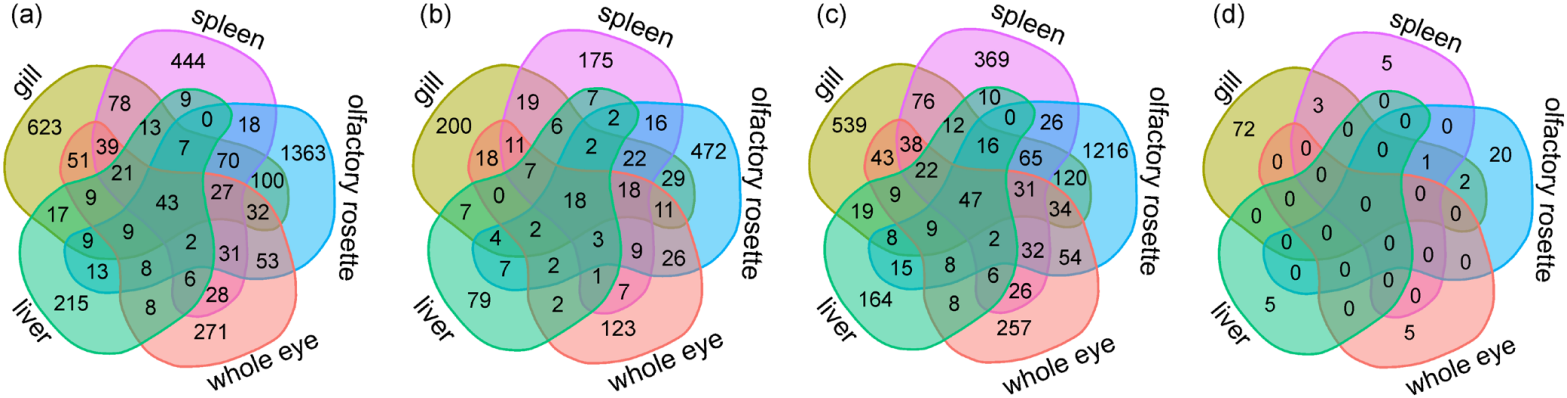
Venn diagrams showing the common and unique (a) *cis*‐eQTLs, (b) eGenes, (c) eSNPs and (d) eGenes&DEGs among the five tissues.

In total, 821 pairs of gene expression‐SNP associations (*cis*‐eQTLs) (268 eGenes, i.e., genes that contain *cis*‐eQTL, and 783 eSNPs, i.e., SNPs, that are significantly associated with eGenes) were observed in the gill, 581 *cis*‐eQTLs (221 eGenes and 537 eSNPs) in the spleen, 1361 *cis*‐eQTLs (489 eGenes and 1294 eSNPs) in the olfactory rosette, 466 *cis*‐eQTLs (198 eGenes and 461 eSNPs) in the eye, and 155 *cis*‐eQTLs (75 eGenes and 154 eSNPs) in the liver (Tables 4 and S6). Approximately 40% to 50% of the *cis*‐eQTLs, eGenes, and eSNPs in each of the four tissues were shared with the others (Figure 4a–c), whereas in the olfactory rosette the proportion of shared *cis*‐eQTLs, eGenes and eSNPs was around 25% (Figure 4a–c). The majority of the overlapped *cis*‐eQTLs (> 99%) had the same direction of regulatory effect (estimated as beta coefficient; Table S6). Around 50% of eGenes were *cis*‐regulated by a single eSNP (46.6% in gill, 55.2% in spleen, 53.4% in olfactory rosette, 54.5% in eye, and 61.3% in liver; Figure S3 and Table S6). Among the identified 953 eGenes, 81 (8.4%) were differentially expressed (eGenes & DEGs) in one or several tissues: 20.5% in the gill, 3.6% in the spleen, 3.3% in the olfactory rosette, 2.0% in the eye, and 2.7% in the liver (Figure 4d, Tables 4 and S6).

**TABLE 4:** The number of *cis*‐ gene‐SNP associations (*cis*‐eQTLs), SNPs with regulatory effects (eSNPs), outlier SNPs found among eSNPs (outlier eSNPs), genes associated with eSNPs (eGenes), differentically expressed genes among eGenes (eGenes&eDEG), eGenes found among candidate genes (genes harbouring outlier SNPs and eGenes) and DEGs found among candidate eGenes (outlier eGenes&DEGs) in different tissues.

Tissue	*cis*‐eQTLs	eSNPs	outlier eSNPs	eGenes	eGenes&DEG	Candidate eGenes	Candidate eGenes&DEG
Gill	821	783	16	268	55	35	6
Spleen	581	537	21	221	8	23	2
Olfactory rosette	1361	1294	31	489	16	66	3
Whole eye	466	461	7	198	4	24	0
Liver	155	154	1	75	2	15	1
Total	2640	2464	52	953	81	128	11

*Note:* The details about outlier SNPs and candidate genes (i.e. genes harbouring outlier SNPs within 5K up‐ and 5K downstream area) can be found in Ozerov et al. (2022).

**FIGURE 5:**
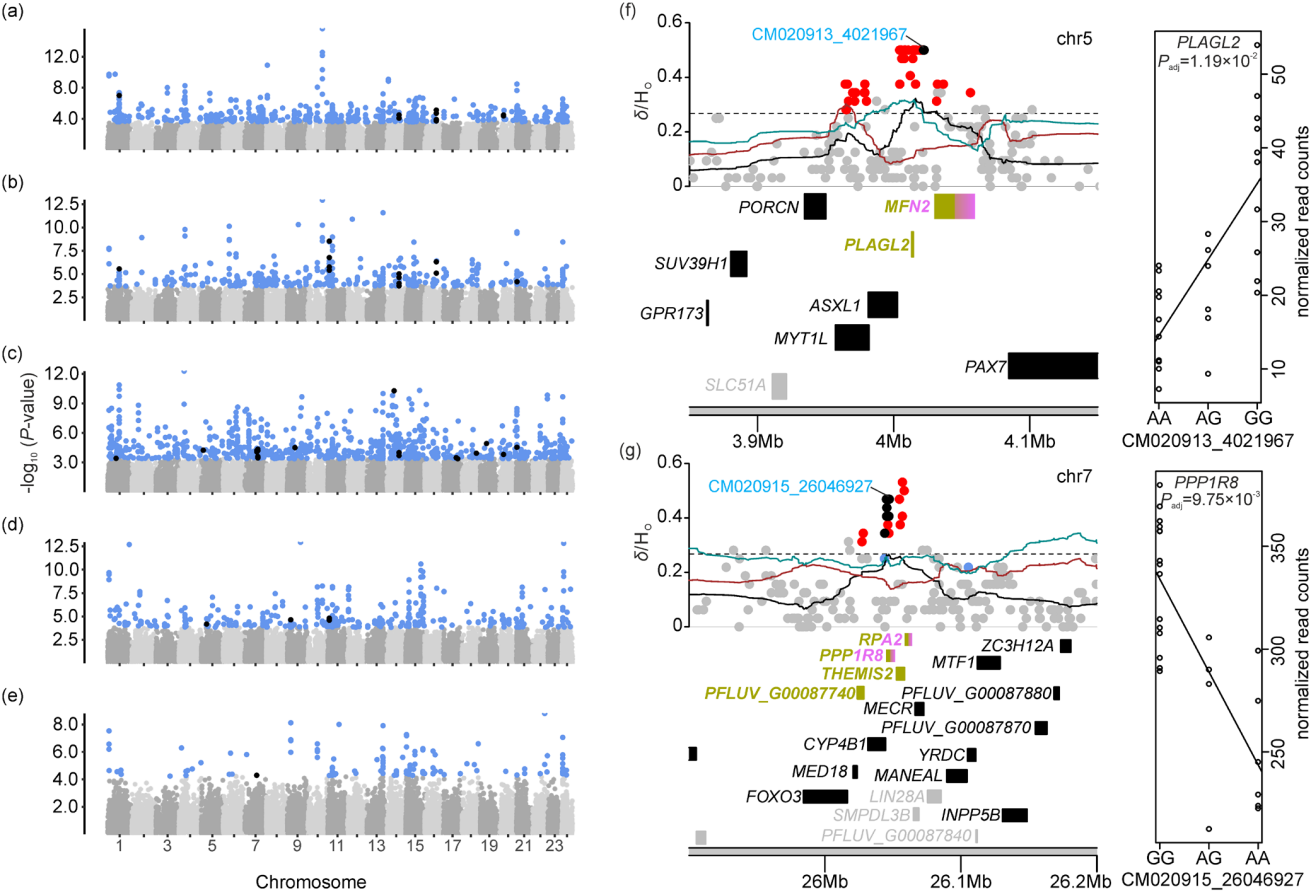
Detected eQTLs and examples of genomic regions potentially involved in adaptive divergence with associated regulatory links. Manhattan plots showing *p*‐values of eSNPs across perch genome in (a) gill, (b) spleen, (c) olfactory rosette, (d) whole eye and (e) liver tissues. Manhattan plots showing example of genomic regions harbouring outlier SNPs of high genetic divergence (*δ*) between perch from humic and clear‐water environment (Ozerov et al. 2022) with significant gene expression‐SNP associations (*cis*‐eQTLs) in (f) *PLAGL2* and (g) *PPP1R8* genes (on the left) and plots showing linear relationships of normalised read counts of the genes with the most significantly associated eSNP genotype (on the right). DEGs are presented by rectangles filled with the colour corresponding to the tissue (gill—olive green, spleen – magenta and olfactory rosette—deep sky blue). Expressed and non‐expressed genes are presented as black‐filled and grey‐filled rectangles, respectively. eSNPs, outlier SNPs, outlier eSNPs, and other SNPs are shown by light blue, red, black, and grey dots, respectively. The colour of the eSNP designation above each Manhattan plot indicates the tissue where *cis*‐eQTLs was detected and corresponds to the tissue colour codes. Moving averages of *δ* and *H*_O_ of perch from humic and clear‐water environment across 50 SNPs are shown with black, brown and cyan solid lines, respectively. Gene symbols are presented as human orthologues and perch GenBank gene IDs (PFLUV_G) for genes with unidentified functions.

The genomic regions of high genetic divergence detected by Ozerov et al. (2022) also harboured eSNPs in one or several tissues (Figure 5). Moreover, outlier SNPs were significantly overrepresented among identified eSNPs in the spleen (Chi‐square test *p*‐value < 0.00001), the olfactory rosette (Chi‐square test *p*‐value < 0.001) and when all tissues were combined (Chi‐square test *p*‐value < 0.001; Table 5). On the other hand, DEGs in one tissue were not always corresponding to the *cis*‐eQTLs for the same tissue. For example, outlier eSNPs associated with the expression of *PLAGL2* (Figure 5a) and *PPP1R8* (Figure 5b) genes in the olfactory rosette tissue, whereas *PLAGL2* and *PPP1R8* were differentially expressed in the gill and the spleen. In addition, three eGenes & DEGs were shared between the gill and the spleen (*IFITM3*—Interferon Induced Transmembrane Protein 3/dispanin subfamily A member 2b‐like, and two uncharacterised genes: PFLUV_G00269580 and PFLUV_G00130350) and one between the gill and olfactory rosette tissues (*CD3E*—T‐cell surface glycoprotein CD3 epsilon chain‐like; Table S6).

**TABLE 5:** Enrichment of outlier SNPs among eSNPs (i.e., SNPs that are significantly associated with gene expression variation) in five tissues based on chi‐square test with Yates correction.

Analyses/Tissue type	Data type	Non‐outlier SNPs	Outlier SNPs	Frequency of outlier SNPs	*p*
RNAseq—gill	eSNPs	783	16	0.0204	0.087
RNAseq—spleen	eSNPs	537	21	0.0391	**< 0.00001**
RNAseq—olfactory rosette	eSNPs	1294	31	0.0240	**0.001**
RNAseq—whole eye	eSNPs	461	7	0.0152	0.809
RNAseq—liver	eSNPs	154	1	0.0065	0.741
RNAseq—five tisssues combined	eSNPs	2464	52	0.0211	**0.001**
WGS—analysis of footprints of selection	SNP data from Ozerov et al. (2022)	800,346	10,245	0.0128	—

*Note:* Significant enrichment is marked with bold.

## 4 Discussion

Genome scans represent an important first step towards uncovering regions involved in adaptation harbouring exceptionally high genetic divergence and/or low diversity patterns suggestive of a selective sweep (Bomblies and Peichel 2022; Ellegren 2014; Storz and Wheat 2010; Vasemägi and Primmer 2005). The main advantage, as well as the limitation, is that the potential candidate regions involved in adaptation can be identified without the incorporation of phenotypic or environmental information. As a result, we frequently lack a detailed understanding of the molecular functions, physiological processes, or phenotypic traits that are involved in adaptation. In this work, we applied a strategy of integrating multiple levels of genomic information using functional genomic and evolutionary approaches, to gain deeper insights into the tissues, molecular functions, genomic regions, genes, and *cis*‐regulatory associations involved in adaptation.

Previous whole genome analyses of Eurasian perch indicated that adaptation to a humic environment may involve a large number of SNPs and hundreds of regions scattered across the genome (Ozerov et al. 2022). Here, we tested whether an integrative strategy would enable us to prioritise the genes and tissues involved in this process. Based on RNA‐seq analyses across five tissues, we detected over 5000 DEGs and prioritised candidate genes containing outlier SNPs based on their expression differences between humic and clear‐water environments. The majority of DEGs were found in the gill, suggesting that ion composition and oxygen levels may be involved in humic acclimation and potentially adaptation in perch. The latter was supported by the significant excess of outlier SNPs among DEGs in the gill, as well as in the spleen, suggesting the involvement of these tissues in adaptation. Thus, by combining adaptive variation with transcriptomic data, we were able to narrow down the initial large list of outlier SNPs by tenfold. Additionally, we found more than two‐and‐a‐half thousand putative *cis*‐eQTLs regulating gene expression, with approximately 8% of genes (81) harbouring *cis*‐eQTLs showing differential expression between humic and clear‐water perch. Furthermore, we observed significant over‐representation of outliers among eSNPs in spleen and olfactory rosette tissues, as well as when combining data from tissues. This suggests that among the identified *cis*‐regulatory links are possible functional variants that have been shaped by selection.

We first detected a significant excess of outlier SNPs among DEGs in the gill and spleen tissues, suggesting their involvement in humic adaptation. Notably, among the five tissues, the gill and spleen also exhibited the highest number of DEGs (*n* = 4311 and *n* = 891, respectively). The observed gene expression variation between humic and clear‐water environments in the gills was anticipated, given that besides their respiratory function, this multifunctional organ is involved in ion exchange, acid–base regulation, osmoregulation, and excretion of nitrogenous waste (Evans et al. 2005). Furthermore, recent work has shown that gill microbial communities and host‐microbe interations may influence the ionoregulatory response in humic environments (François‐Étienne et al. 2023). We found that the majority of genes involved in the transmembrane transportation of ions, such as solute carriers (*SLC*s), calcium channel subunits (*CACN*s), potassium channel subunits (*KCN*s) and sodium channel subunits (*SCN*s), were differentially expressed exclusively in the gills (ranging from 50% to 85%; Table S3). This is in agreement with the differences in ion composition between humic and clear‐water lakes due to variation in DOC concentrations (Arvola et al. 2010; Erlandsson et al. 2010) and the function of the gill epithelium serving as a barrier between the blood and the aquatic environment (Evans et al. 2005). In addition, our previous whole‐genome scan for footprints of selection identified a substantial number of candidate genes involved in the transportation of Na^+^/H^+^, K^+^ and Ca^2+^ ions and the maintenance of pH balance (Ozerov et al. 2022). Similarly, these gene families have been shown to play a role in adaptation to acidic (Haenel et al. 2019) and alkaline (Xu et al. 2017) habitats in other fish species.

In addition to the gill, we observed a significant excess of outliers among DEGs expressed in the spleen, which also exhibited the second highest number of DEGs. The fish spleen functions as a secondary lymphoid organ, where adaptive immune responses are generated, and it is an important source of immunoglobulins in teleosts (Bjørgen and Koppang 2021; Fänge and Nilsson 1985; Flajnik 2018). In line with this, the majority of enriched GO terms for spleen DEGs were related to immune system functioning (Table S4). Interestingly, our earlier work identified drastic differences in diplostomid parasite communities between humic and clear‐water perch (Noreikiene et al. 2020). Due to the effect of humic substances on whole aquatic communities, including underwater vegetation and zooplankton (Blanchet et al. 2022), the abundance of free‐living trematode parasites and their intermediate hosts, gastropods (Selbach et al. 2020) in humic lakes is much lower compared to that in the clear‐water lakes (Halmetoja et al. 2000; Pietrock and Marcogliese 2003). Consequently, the high prevalence of eye flukes in clear‐water lakes along with the absence of diplostomid parasites in humic lakes (Noreikiene et al. 2020) may be associated with the elevated number of immune response‐related DEGs in the spleen. In addition, the spleen plays an active role in haemopoiesis in fishes, being a primary site for the destruction of aged red cells and the recycling of iron (Fänge and Nilsson 1985). Given that humic lakes show highly stratified temperature and oxygen levels, where hypoxia can occur even at a depth of a few meters (Karpowicz and Ejsmont‐Karabin 2018; Karpowicz et al. 2023), it is plausible that the processes of haemopoiesis and iron recycling in perch are interconnected with these hypoxic conditions in humic lakes.

Regulatory polymorphisms that shape gene expression are known to play a key role linking genetic variation to organismal performance and phenotypic traits (Cano‐Gamez and Trynka 2020; Li and Ritchie 2021; Williams et al. 2007). Recent efforts to uncover associations between regulatory mechanisms and disease traits have shed light on the genetic control of gene expression in humans and farmed animals (Clark et al. 2020; GTEx Consortium 2020; GTEx Consortium et al. 2015; Kim‐Hellmuth et al. 2020; Liu et al. 2022). However, for the majority of non‐model organisms, gene regulation mechanisms and their connections to phenotypic traits remain to be characterised (Krishnan et al. 2022; Marand et al. 2023). Here, we identified 2640 *cis*‐eQTLs that are associated with gene expression variation in perch, of which 50%–70% were tissue‐specific. This finding aligns with previous observations of localised effects of *cis*‐eQTLs on gene expression, often within a particular tissue or cell type (Hill et al. 2021; Signor and Nuzhdin 2018). Furthermore, the significant over‐representation of outlier SNPs among detected eSNPs in the spleen and olfactory rosette tissues, as well as when considering all tissues combined, suggests that some of the *cis*‐acting regulatory associations are likely involved in the humic adaptation of perch. Our results, therefore, corroborate previous studies that underline the importance of *cis*‐regulation in adaptive evolution (Signor and Nuzhdin 2018). For example, a recent analysis of temperate and tropical house mice in different environments found that *cis*‐regulatory changes are likely mechanisms for adaptive body size evolution (Ballinger et al. 2023). Similarly, an intraspecific comparison between marine and freshwater three‐spine sticklebacks showed that *cis*‐eQTLs drive expression variation (Verta and Jones 2019).

To pinpoint the most promising candidate genes involved in humic adaptation, we aligned the identified *cis*‐eQTLs with the genomic regions showing the strongest signals of selection harbouring DEGs. Among the putative regulatory links involved in humic adaptation, we observed *cis*‐eQTLs involving DEGs within several candidate regions (e.g., chr. 8, CM020916_12156238—*IFITM3*; chr. 14, CM020922_15798921—*TRIM39*; and chr. 7, CM020915_23598807—*TCHH*). For example, interferon‐induced transmembrane protein 3 (*IFITM3*) is a member of the IFITM family, which is composed of important innate immune effectors involved in protecting against diverse viral infections in vertebrates (Spence et al. 2019). *IFITM3* was consistently down‐regulated in the gill, spleen and olfactory rosette of humic perch, which may reflect the negative effect of DOC and low pH on viral communities in humic lakes (Blanchet et al. 2022; Dalziel et al. 2016). Another notable association involved trichohyalin (*TCHH*), a versatile protein found in hair follicles and other specialised epithelial tissues of mammals, that assists in mechanical strengthening and in connecting the protective outer layers of cells to the inner structural framework made of keratin filaments (Steinert et al. 2003). In contrast to terrestrial animals, the fish epidermis does not have a dead, keratinised surface (Rakers et al. 2010). The *TCHH*‐like gene was up‐regulated in multiple tissues of humic perch, which, together with its function in maintaining skin structures, may indicate its involvement in helping perch to thrive in a highly acidic humic environment.

In addition, we detected several *cis‐*eQTLs in the regions of high genetic divergence between humic and clear‐water perch harbouring DEGs on chromosomes 5 and 7 (Figure 5). DEGs located in these regions are involved in multiple functions, such as the regulation of anatomical structure development (*PLAGL2*), regulation of oxidative stress response (*PLAGL2*), mitochondrial fusion (*MFN2*), response to stress (*RPA2*, *THEMIS2*) and glycogen metabolism (*PPP1R8*). Notably, given its regulatory function in oxidative stress response (Guo et al. 2007), the up‐regulation of *PLAGL2* in the gills of perch living in humic lakes may be linked to the hypoxic conditions. Similarly, the down‐regulation of *PPP1R8* in the gills and spleen of humic perch may indicate divergent energy metabolism in humic versus clear‐water perch, as high DOC concentration reduces the diversity of aquatic prey communities in humic lakes (Blanchet et al. 2022). However, the relatively low number of DEGs found in the liver together with the lack of enrichment of outlier SNPs suggests that energy metabolism probably does not play a major role in the adaptation to a humic environment. It is also important to note that we did not quantify sex‐specific differences in gene expression, and further research is needed to investigate how sex‐biased gene expression varies across different tissue types. Similarly, an important unresolved question is how the level of gene expression influences the selective pressures acting on regulatory regions (Joshi et al. 2021; Wolf et al. 2023).

In conclusion, we employed an integrative genomics approach to gain further insights into the tissues, molecular functions, genomic regions, genes, and putative regulatory associations involved in the adaptation of a keystone freshwater fish. This integrative strategy enabled us to prioritise candidate genes under selection based on their expression patterns, and to identify tissues and organs that potentially play a key role in adaptation. Furthermore, we identified putative causative links between SNPs and transcript abundance variation, and found that some of the *cis*‐acting regulatory associations have likely been shaped by divergent selection linked to humic substances. Thus, although we did not explicitly disentangle the plastic and genetic effects on gene expression variation by studying organisms in their native environment, this study successfully bridged the gap between genome scans for selection and functional genomics approaches. We expect that the framework combining evolutionary genetics and functional omics fields will serve as a valuable guide for increasing our understanding of the regulatory mechanisms and genetic basis of phenotypic diversification and adaptation in a wide range of species.

## Author Contributions

Anti Vasemägi and Riho Gross devised and planned the study. Mikhail Ozerov conducted the bioinformatics and population genomic analyses. Mikhail Ozerov and Anti Vasemägi collaborated in writing the initial draft of the manuscript. Anti Vasemägi, Kristina Noreikiene and Konrad Taube participated field sampling. Kristina Noreikiene and Konrad Taube contributed to laboratory work. All authors participated in revising and refining the manuscript.

## Conflicts of Interest

The authors declare no conflicts of interest.

## Supporting information


**Figure S1.** MDS plots showing relationships of (a) 30 gill, (b) 31 spleen, (c) 29 olfactory rosette tissue samples. Excluded samples (see Material and Methods) are marked with circles.
**Figure S2.** Venn diagram showing the common and unique genes expressed in each of the five tissues.
**Figure S3.** Enrichment of outlier SNPs among DEGs. Histogram of a permutation distribution of the difference of the frequency of outlier SNPs in DEGs and in non‐DEGs in all tissues, gill, spleen, olfactory rosette, whole eye and liver. Vertical red line shows the observed difference. The test was based on 1000 permutations.
**Figure S4.** Summary statistics of the *cis*‐genetic variants associations and gene expression levels. *p*‐value distributions of all the *cis*‐associations detected in (a) gill, (b) spleen, (c) olfactory rosette, (d) whole eye and (e) liver. Distributions of numbers of eSNP per eGene in (f) gill, (g) spleen, (h) olfactory rosette, (i) whole eye and (j) liver.

**Table S1.** Sampled individuals of *P. fluviatilis* , their location, date of sampling, sex, total length (TL) and sampled tissues.
**Table S2.** Genetic relationships among studied *P. fluviatilis* populations, estimated as Nei’s genetic distances (Nei 1978; above diagonal) and pairwise *F*_ST_ (below diagonal).
**Table S3.** Differentially expressed genes (DEGs) in *P. fluviatilis* between humic and clear‐water environment found in five studied tissues and the number of previousely detected outlier SNPs (see Ozerov et al. 2022) per DEG. Cells without reported log2‐fold change indicate the absence of differential expression.
**Table S4.** List of gene ontology (GO) terms with significant enrichment among the differentially expressed genes in *P. pluviatilis* (N—is the total number of genes; B—is the total number of genes associated with a specific GO term; n—is the number of genes in the top of the input list; b—is the number of genes in the intersection; Enrichment = (b/n)/(B/N)).
**Table S5.** Enrichment and depletion of outlier SNPs among differentially expressed genes (DEGs) in each annotation category. Significant *p*‐values (≤ 0.05) are highlighted in bold.
**Table S6.** Significant *cis*‐gene‐SNP associations (*cis*‐eQTLs), SNPs with regulatory effects (eSNPs), associated genes (eGenes), and regulatory effect (beta) in studied tissues. Differentically expressed genes among eGenes (eGene&DEG), outlier SNPs among eSNPs (outlier eSNPs), eGenes found among candidate genes (genes harbouring outlier SNPs and regulatory SNPs, candidate eGene), and DEGs found among candidate eGenes (candidate eGene&DEG) are marked with “X”. The details about outlier SNPs and candidate genes (i.e., genes harbouring outlier SNPs within 5K up‐ and 5K downstream area) can be found in Ozerov et al. (2022).

Data S1.

## Data Availability

Raw sequence reads have been deposited in the NCBI Sequence Read Archive (SRA) under BioProject accession number PRJNA1014212 (SRR25949659—SRR25949804). A custom R script and accompanying dataset to perform a permutation test to determine if DEGs and nearby regions are enriched for outlier SNPs are available at figshare portal: DOI: https://doi.org/10.6084/m9.figshare.27266334.
