# Supplementary material for: Integrative Genomics Refines Tissues, Candidate Genes and Putative Regulatory Links Involved in the Humic Adaptation of Keystone Freshwater Fish
Source: Mol Ecol. 2025 Feb 18;34(15):e17698. doi: 10.1111/mec.17698 (PMC12288802; doi:10.1111/mec.17698)
Supplement: Supplementary file 1 — Figure S1. MDS plots showing relationships of (a) 30 gill, (b) 31 spleen, (c) 29 olfactory rosette tissue samples. Excluded samples (see Material and Methods) are marked with circles. Figure S2. Venn diagram showing the common and unique genes expressed in each of the five tissues. Figure S3. Enrichment of outlier SNPs among DEGs. Histogram of a permutation distribution of the difference of the frequency of outlier SNPs in DEGs and in non‐DEGs in all tissues, gill, spleen, olfactory rosette, whole eye and liver. Vertical red line shows the observed difference. The test was based on 1000 permutations. Figure S4. Summary statistics of the cis‐genetic variants associations and gene expression levels. p‐value distributions of all the cis‐associations detected in (a) gill, (b) spleen, (c) olfactory rosette, (d) whole eye and (e) liver. Distributions of numbers of eSNP per eGene in (f) gill, (g) spleen, (h) olfactory rosette, (i) whole eye and (j) liver. [file MEC-34-e17698-s001.docx]

*Supplementary figures for*:

**Integrative genomics refines tissues, candidate genes and putative regulatory links involved in the humic adaptation of keystone freshwater fish**

Ozerov M.Yu.^1,*^, Noreikiene K.^2,3^, Taube K.^2^, Gross R.^2^, Vasemägi A.^2,4,*^

^1^Biodiversity Unit, University of Turku, Vesilinnantie 5, 20500 Turku, Finland

^2^Chair of Aquaculture, Estonian University of Life Sciences, Kreutzwaldi 46a, 51014 Tartu, Estonia

^3^Institute of Biosciences, Life Sciences Center, Vilnius University (Vilnius, Lithuania)

^4^Swedish University of Agricultural Sciences, Sötvattenslaboratoriet, Stångholmsvägen 2, 17893 Drottningholm, Sweden

^*^Contributed equally.

**Corresponding author**: Anti Vasemägi, Sötvattenslaboratoriet, Stångholmsvägen 2, 17893 Drottningholm, tel. +46104784277, anti.vasemagi@slu.se

**Supplementary figures S1-S4**


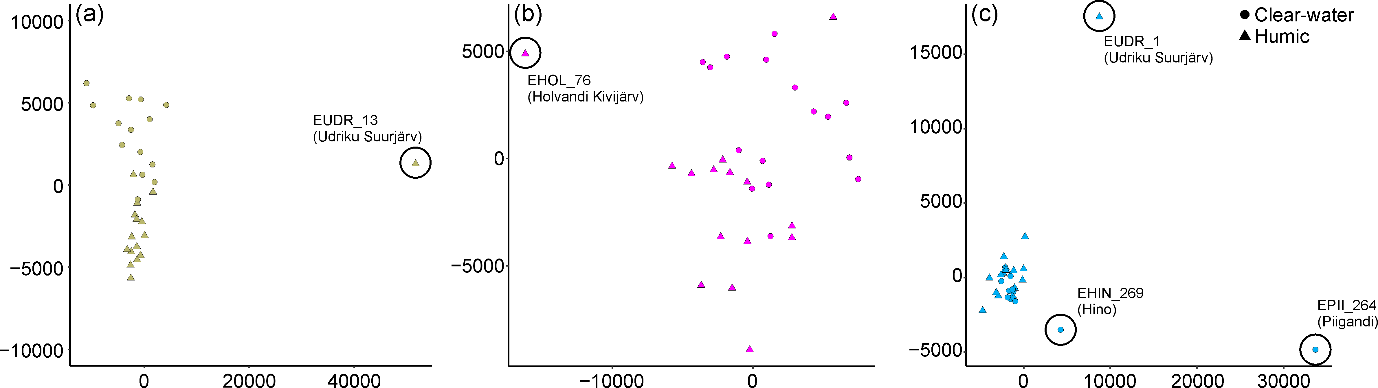


**Fig. S1.** MDS plots showing relationships of (a) 30 gill, (b) 31 spleen, (c) 29 olfactory rosette tissue samples. Excluded samples (see Material and Methods) are marked with circles.


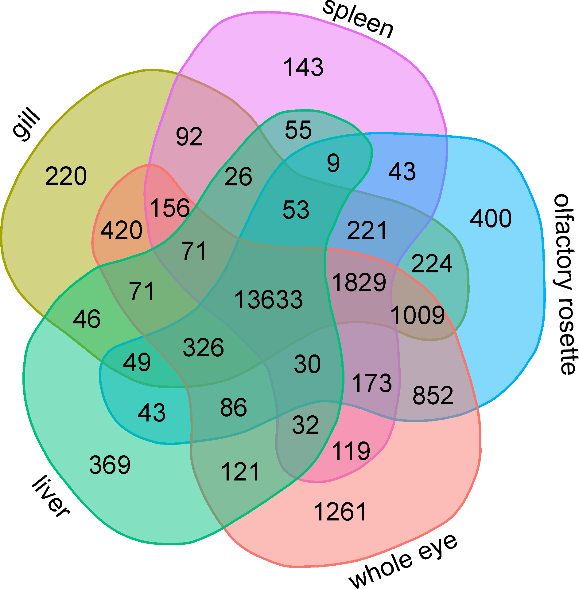


**Fig. S2.** Venn diagram showing the common and unique genes expressed in each of the five tissues.

**
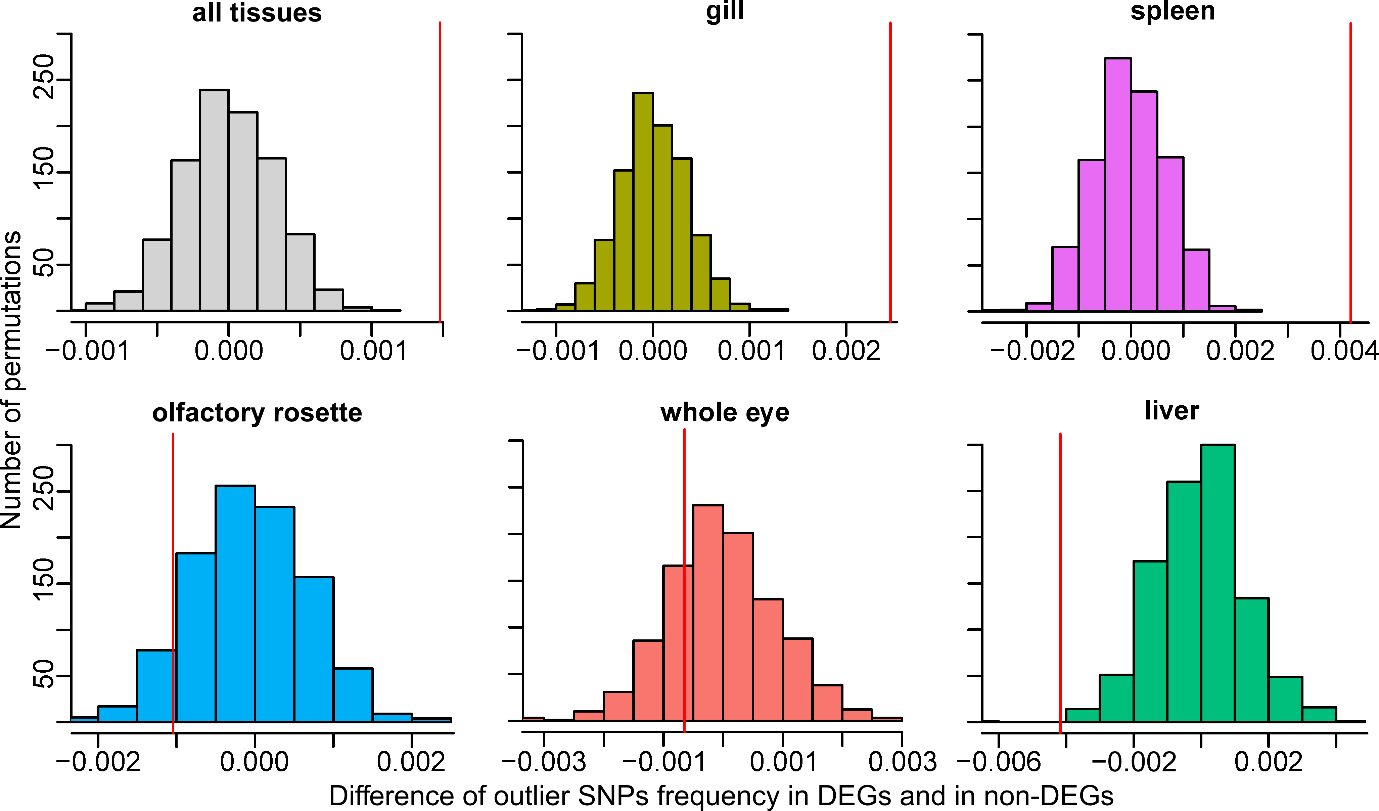
**

**Fig. S3.** Enrichment of outlier SNPs among DEGs. Histogram of a permutation distribution of the difference of the frequency of outlier SNPs in DEGs and in non-DEGs in all tissues, gill, spleen, olfactory rosette, whole eye and liver. Vertical red line shows the observed difference. The test was based on 1000 permutations.

**

**

**Fig. S4.** Summary statistics of the *cis*-genetic variants associations and gene expression levels. *P*-value distributions of all the *cis*-associations detected in (a) gill, (b) spleen, (c) olfactory rosette, (d) whole eye and (e) liver. Distributions of numbers of eSNP per eGene in (f) gill, (g) spleen, (h) olfactory rosette, (i) whole eye and (j) liver.
